# Colloidal Light Scattering Distorts Fluorometric Quantification of mRNA in Lipid Nanoparticles

**DOI:** 10.3390/cimb48080805

**Published:** 2026-08-09

**Authors:** Agnieszka Klusek, Kamil Adasiewicz, Andżelika Bystrzejewska, Piotr J. Rudzki, Maciej Wieczorek, Ewelina Juszczyk

**Affiliations:** Celon Pharma S.A., Research and Development Center, 15 Marymoncka, 05-152 Kazuń Nowy, Poland

**Keywords:** drug delivery, fluorescence, pharmaceutical development, lipid nanoparticles (LNP), messenger RNA (mRNA), physical chemistry, analytical chemistry, photochemistry, nanoparticles

## Abstract

Accurate measurement of messenger RNA (mRNA) concentration and encapsulation efficiency remains a challenging task during mRNA-lipid nanoparticle (LNP) formulation development and quality control. RiboGreen-based fluorescence assays are often used for this purpose. LNP formulations are colloidal systems and may exhibit turbidity, which can interfere with fluorescence detection. The impact of LNP-related optical properties on assay performance remains insufficiently characterized. This study aimed to evaluate whether and how physicochemical factors associated with LNP-based drug-delivery systems influence fluorometric mRNA quantification. Empty LNPs, with the same lipid composition as mRNA-loaded LNPs, were used as a model matrix causing turbidity. Their effect on the RiboGreen fluorescence signal was assessed following dilution and then in different scenarios in the presence of free and encapsulated mRNA. The UV–Vis spectrum of the placebo dispersion was recorded to examine possible optical interference in the measurement range. We quantitatively characterized the suppression of fluorescence signals in the presence of LNPs in a concentration-dependent manner. These findings indicate that turbidity and LNP-associated matrix effects can bias RiboGreen-based mRNA quantification. To ensure reliable determination of mRNA concentration and accurate calculation of encapsulation efficiency in LNP formulations, method-specific dilution factors should be applied.

## 1. Introduction

Messenger RNA (mRNA)-based therapeutics have moved rapidly from an experimental concept to clinical practice. By enabling transient protein expression without genomic integration, mRNA offers a flexible approach for prophylactic vaccines, cancer immunotherapy, and protein replacement [1,2]. This progress has depended not only on RNA sequence design, but also on delivery technologies that protect mRNA from rapid enzymatic degradation, support cellular uptake, and enable cytoplasmic release. Free mRNA is poorly suited for direct administration because of its polyanionic character and susceptibility to enzymatic degradation. Among non-viral delivery systems, lipid nanoparticles (LNPs) have become the leading platform for systemic mRNA delivery [1,2,3]. Typical mRNA-LNP compositions contain an ionizable lipid, helper phospholipid, cholesterol, and polyethylene glycol (PEG)-modified lipid [4]. Together, these components drive particle formation, RNA encapsulation, colloidal stability, plasma circulation behavior, and delivery performance. 

The quality and biological activity of mRNA-LNP formulations are closely linked to their physicochemical properties. Critical quality attributes (CQAs), including particle size distribution, polydispersity index (PDI), surface charge, mRNA loading, encapsulation efficiency (EE%), and colloidal stability, are therefore routinely assessed during formulation development [3,5,6,7]. Among these parameters, mRNA concentration and EE% are particularly important because they affect dose calculation, batch-to-batch comparison, stability assessment, and interpretation of biological activity data. They can also be employed as process-end indicators (in-process control) during mRNA-LNP product manufacturing. Analytical methods used for these measurements should therefore be sensitive and reproducible [8], but also compatible with the colloidal lipid matrix. Application of recent UV–vis methods is limited due to their up to 1000-fold lower sensitivity [9,10,11] and by the interference from formulation excipients or detergents at 260 nm wavelength [10]. Fluorescence-based nucleic acid assays are widely used for RNA quantification due to their sensitivity, simplicity to perform, and small-sample-volume requirements. RiboGreen and related nucleic acid-binding dyes generate strong fluorescence after binding to nucleic acids and can be measured using standard fluorescence plate readers or fluorometers [12,13]. This makes RiboGreen-based assays practical for formulation screening, process optimization, and stability testing, where multiple samples need to be quantified with limited material availability. In mRNA-LNP characterization, RiboGreen assays are commonly used to estimate accessible RNA, total RNA, and EE% [13,14]. In a typical workflow, intact LNPs are incubated with the dye to quantify non-encapsulated or externally accessible mRNA. A second aliquot is treated with a detergent or surfactant, most often Triton X-100, to disrupt or solubilize the nanoparticle structure and allow the dye to bind to RNA previously unavailable due to their enclosure within the LNPs [14]. Encapsulated RNA is calculated as the difference between total and accessible RNA, and EE% is expressed as a percentage fraction of that difference to total RNA content.

Nanoparticle-related assay interference is a known issue in optical and spectroscopic measurements. Nanoparticles may affect readouts through scattering, intrinsic absorbance or fluorescence, interactions with assay reagents, and matrix-dependent changes in assay behavior [15,16,17]. For mRNA-LNPs, the same colloidal matrix that protects and delivers mRNA is present during fluorometric quantification. A change in fluorescence intensity should therefore not be automatically interpreted as a change in RNA concentration or EE%. Part of the signal change may originate from the optical properties of the nanoparticle dispersion itself. Although RiboGreen assays are routinely used in mRNA-LNP development, the contribution of LNP optical properties to the fluorescence signal remains insufficiently studied. The relative impact of colloidal light scattering, excitation/emission attenuation, dye–LNP interactions, and RNA accessibility on apparent mRNA concentration has not been systematically deconvoluted in opalescent LNP dispersions. Addressing this knowledge gap is especially relevant in the workflow of LNP formulation development, as inaccurate RNA quantification can affect batch comparison, lead to inconclusive stability assessment and incorrect interpretation of LNP quality attributes, and, in consequence, influence the patient’s safety.

The aim of this study was to evaluate the optical and physicochemical interference of LNP matrix components on fluorometric determination of mRNA concentration by the RiboGreen assay. This study focuses on colloidal light scattering and optical attenuation in opalescent LNP dispersions while also considering dye-related effects and RNA accessibility to dye under intact and altered assay conditions. To isolate optical effects, we applied a novel methodological approach utilizing placebo containing LNPs, with the same lipid composition as in the studied delivery system containing mRNA, as a model matrix causing turbidity. We also propose a novel method-specific solution to increase the reliability of mRNA analytical quantification. 

## 2. Materials and Methods

CleanCap ^®^ FLuc mRNA (5 moU) at 1 mg/mL was purchased from TriLink BioTechnologies (San Diego, CA, USA). RNAse-free water, 20X TE, RNAse-free, Quant-iT™ RiboGreen^®^ Assay Kit and 96-well plates were purchased from Thermo Fischer Scientific (Waltham, MA, USA). Triton™ X-100 was purchased from Sigma-Aldrich (Saint Louis, MO, USA). PBS 20X was purchased from EURx (Gdańsk, Poland). LNP formulation and placebo were manufactured by Celon Pharma S.A. (Kazuń Nowy, Poland). All measurements were performed using Glomax Discover Microplate Reader purchased from Promega (Madison, WI, USA). Placebo’s UV–vis measurement was performed using a V-750 Double Beam UV-Visible Spectrophotometer purchased from JASCO Inc. (Hachioji, Japan). A plate shaker-thermostat was purchased from Biosan (Riga, Latvia). A thermal shaker heater was purchased from Hettich (Kirchlengern, Germany).

### 2.1. Preparation of Reagents

20X TE buffer and 20X PBS were diluted 1:19 (*v*/*v*) with RNase-free water in an RNase/DNase-free polypropylene tubes. Triton X-100 was diluted 1:4 (*v*/*v*) with RNase-free water in an RNase/DNase-free polypropylene tubes and then 1:19 (*v*/*v*) with 1X TE buffer. RiboGreen was diluted with 1X TE buffer 1:199 (*v*/*v*). Solutions were protected from light and used directly after preparation.

### 2.2. Preparation of Standards/Samples

The mRNA standard was removed from a refrigerator, incubated for 3 min at 37 °C, mixed gently and incubated for the next 3 min at 37 °C. The amount of 100 µL of the standard was mixed with 900 µL of 1X TE. Then, 40 µL of obtained solution was transferred to 160 µL of 1X TE and mixed gently. The final nominal concentration was 20 µg/mL.

### 2.3. The 96-Well Plate Preparation and Pipetting Workflow

All samples were treated with Triton X-100 before RiboGreen addition to disrupt the LNP structure, minimize potential post hoc association of free mRNA with empty LNPs, and avoid variability related to detergent concentration. Triton 1% was added using the reverse pipetting method.

To well A, 250 µL of solution was added in a sample (20 μg/mL):TE ratio of 1:4 (*v*/*v*). A total of 200 μL of the solution was added to the remaining wells (B–E). To wells B and C, the following *v*/*v* ratio was added: TE:Triton:placebo:sample from row A = 1:3:3:3. To wells D and E, the following *v*/*v* ratio was added: TE:Triton:sample from row A = 2:5:3. The samples were incubated for 3 min at 37 °C, shaken for 10 s/1000 rpm. Afterwards, 100 µL of prepared RiboGreen solution was added, again incubated for 3 min at 37 °C, and shaken for 10 s/1000 rpm. Immediately after incubation, the plate was read using 485 nm excitation wavelength and 500–550 nm emission wavelengths. 

### 2.4. Statistical Methods

Datasets in experiment 1 were compared using an unpaired *t*-test with Welch’s correction at significance level α = 0.05 (GraphPad Prism version 7.05, GraphPad Software, Boston, MA, USA).

## 3. Results

We used placebo (i.e., empty lipid nanoparticles composed of a proprietary ionizable lipid, phospholipid, sterol, and PEG-lipid, without mRNA) as model matrix causing turbidity. This study consisted of two complementary experiments. Initially, the influence of various amounts of placebo on the fluorescence signal for a fixed amount of mRNA standard was evaluated. Then, the effect of an additional amount of placebo was studied in different scenarios for samples containing free or/and encapsulated mRNA. Additionally, to investigate the potential interference of placebo components with the fluorometric assay, the UV–vis spectrum of the placebo was recorded. 

### 3.1. Effect of Various Amounts of Placebo on Measurement of Fixed mRNA Concentration

**Figure 1:**
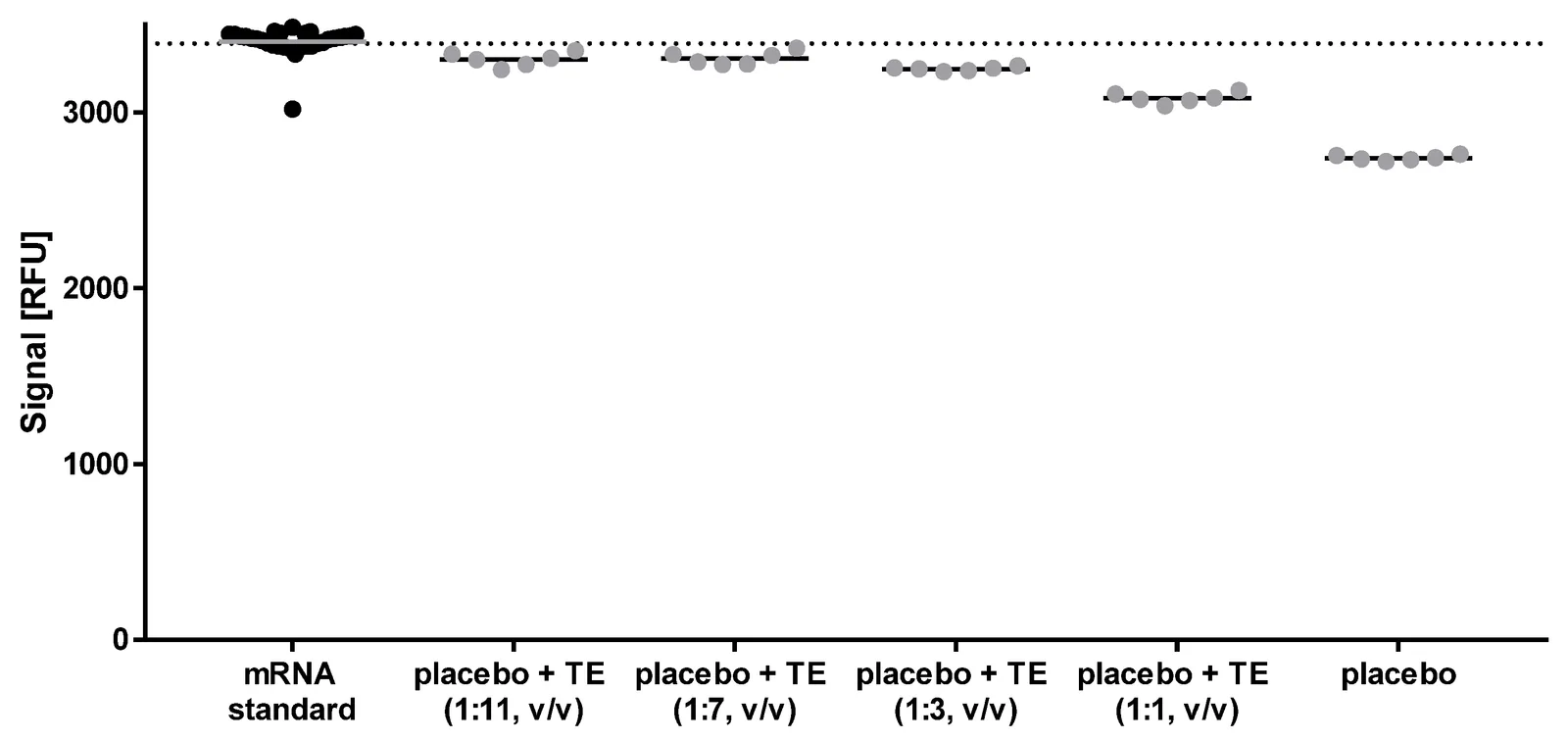
Scatterplot for analytical signal in samples containing a fixed amount of mRNA standard and different amounts of placebo decreasing from left (100%) to right (0%). Gray dots indicate samples containing placebo, and black dots indicate samples without placebo (the reference). Horizontal lines indicate the mean signal. TE = Tris-EDTA buffer.

The mRNA concentration was fixed at 20 μg/mL and combined with different amounts of placebo in order to evaluate its effect on the fluorescence signal. The mRNA without placebo was used as a reference. Its concentration was calculated based on the theoretical amount of mRNA added to the sample and adjusted with TE to the corresponding concentration expressed as a *v*/*v* ratio. Individual results for samples with various amounts of placebo were plotted on Figure 1, while summary statistics are presented in Table 1.

The experiment revealed good measurement repeatability (RSD < 2.4%) and a clear trend of the analytical signal being inversely proportional to the amount of placebo added. Although mRNA concentration was fixed (20 μg/mL—based on the development of the method, it was determined as suitable for routine analyses conducted at our company), we observed a 19.42% decrease in the signal in the sample containing placebo compared to the reference sample containing mRNA without placebo. The sample with placebo diluted 1:1 was also outside of the company’s ±5% acceptance criteria for relative difference set in this experiment (see the footnote to Table 1). Despite a statistically significant decrease in signal (*p* < 0.05, Table 1), placebo diluted 1:3 was just inside the acceptance criteria and further dilution resulted in acceptable results.

### 3.2. Effect of Additional Amount of Placebo in Various Scenarios

**Figure 2:**
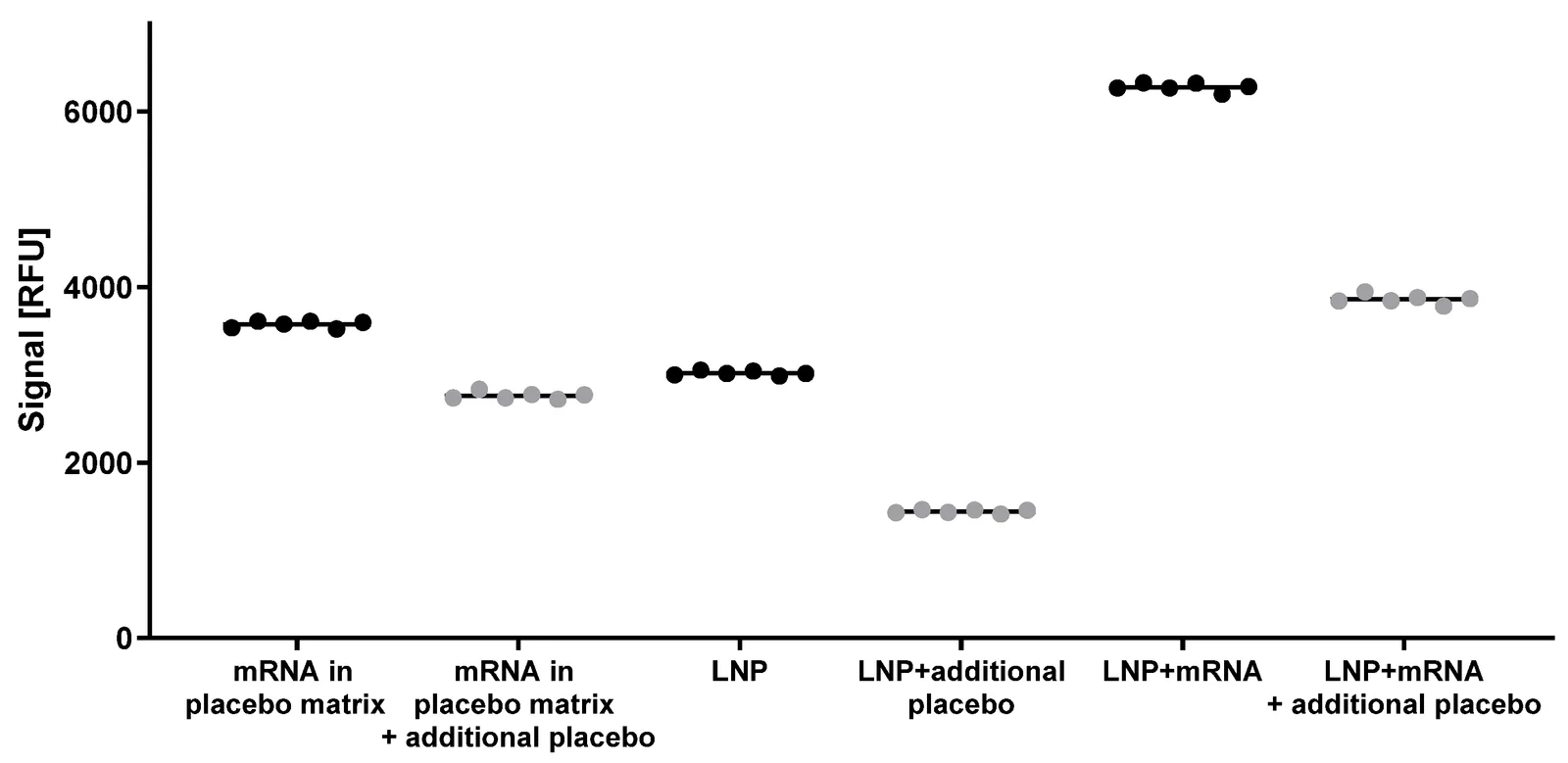
Scatterplot for analytical signal in reference samples (black dots) and samples with additional placebo (grey dots). Horizontal lines indicate mean signal.

After confirming the placebo influence on signal intensity in the first experiment, we aimed to mimic various scenarios possible in laboratory practice. The second experiment included measurements of samples with and without additional placebo. The placebo refers to unloaded LNPs with the same lipid composition as the mRNA-LNP formulation. The ratio of placebo amount in samples with and without additional placebo (a concentration was calculated based on how many mRNA would theoretically be added to the sample and diluted to a concentration of 20 μg/mL) was 503:3 (*v*/*v*). The additional placebo was prepared by diluting 30 uL of placebo with 970 μL of TE, ensuring a critically high amount of possibly interfering compounds. The following scenarios were evaluated: placebo (baseline), placebo + mRNA standard (mimicking free mRNA), LNP containing encapsulated mRNA (encapsulation efficiency was 98%—data obtained from different study), and LNPs containing encapsulated mRNA mixed with mRNA standard (free and encapsulated mRNA together). Individual results for various laboratory samples used in this experiment are plotted in Figure 2, while summary statistics are presented in Table 2.

The results are consistent with the previous experiment. Repeatability of measurements (RSD < 2.3%) was not influenced by the addition of placebo (F test, *p* > 0.05). In each scenario, suppression of signal was observed in samples containing an additional amount of placebo (*p* < 0.05, Table 2), with greater suppression observed in the mRNA-LNP sample (−52.50%) rather than for free mRNA (−22.72%). 

### 3.3. UV–Vis Spectrum of Placebo

**Figure 3:**
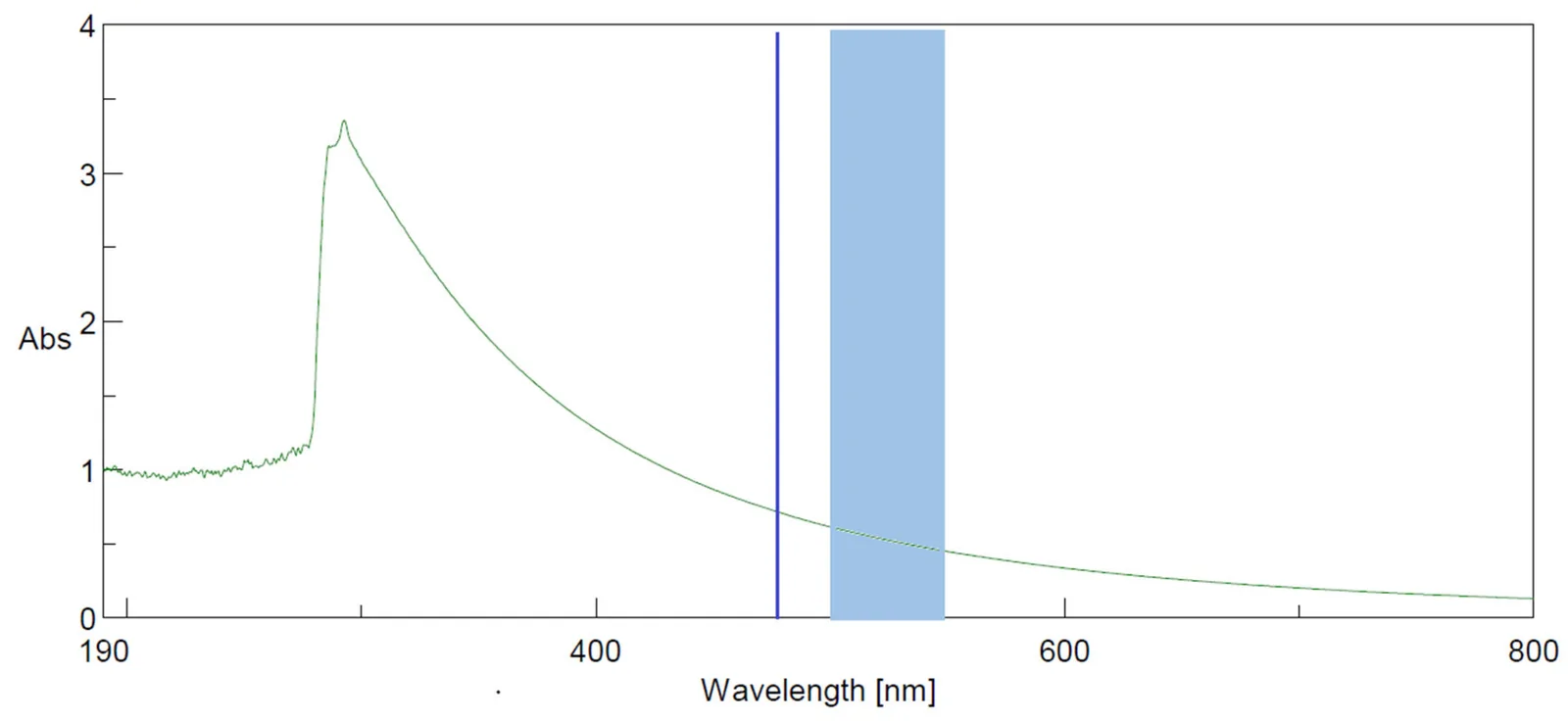
The UV–vis absorption spectrum of the placebo solution diluted (1:10); RiboGreen assay excitation wavelength: 485 nm; emission from 500 to 550 nm.

The UV–vis spectrum was recorded for the placebo solution diluted 1:10 with PBS (Figure 3). The measured absorbance ranged from 0.5 to 1.0 within the spectral region corresponding to the assay excitation wavelength (485 nm) and emission range (500–550 nm), indicating optical attenuation in this region. These values reflect overall optical attenuation and do not distinguish between absorption and scattering.

## 4. Discussion

The presence of light-scattering particles, such as lipid nanoparticles, may cause interference with fluorescence detection, and therefore quantification, of mRNA by attenuating the excitation light reaching the fluorophore and by scattering or partially absorbing the emitted fluorescence signal. These optical effects, including the inner filter effect (IFE) [15], may result in reduced signal intensity, increased measurement variability, and deviations from the linear relationship between fluorescence intensity and analyte concentration. Up to now, this problem has been insufficiently described in the literature and lacks specific quantitative results and standardized methodologies. Our study provides quantitative data and introduces an experimental advancement by using LNP-containing placebo as the model matrix causing turbidity. Instead of using the general term “matrix effects”, we focus specifically on turbidity as a possible cause of analytical problems. Finally, we propose applying method-specific dilution factors to ensure reliable determination of mRNA concentration by the RiboGreen assay and accurate calculation of encapsulation efficiency in LNP-based drug-delivery systems.

The first experiment showed a relevant suppression of the fluorescence signal (i.e., relative difference ≤ 5%) for placebo diluted in ratios 1:3 or more (*v*/*v*) compared to the signal for the reference mRNA standard. As the dilution increased, a progressive decrease in the differences between successive measurement points was observed, eventually reaching a plateau (Figure 2). This effect may be attributed to light absorption and/or scattering by the placebo components within the excitation and emission wavelength ranges, as indicated by the UV–vis spectrum presented in Figure 3. Due to the turbid nature of the sample, the application of a method-specific dilution factor is therefore a key step of the analytical procedure prior to fluorescence measurement.

The second experiment showed that samples with additional placebo produced much lower fluorescence signals than the corresponding mRNA reference standard solutions alone. This effect was most pronounced for the mRNA-containing LNP sample, in which the signal was markedly lower (−52.20%, Table 2) than that obtained for the reference sample containing the same nominal mRNA concentration without placebo. Because the placebo consists of empty LNPs with the same lipid composition as the mRNA-loaded LNPs, the observed decrease should be interpreted as an effect of the LNP matrix rather than as an effect of placebo alone. The data suggest that the lipid nanoparticle dispersion can attenuate or otherwise disturb the RiboGreen fluorescence response, most likely through a combination of light scattering, optical attenuation within the excitation and emission ranges, and matrix-related effects on dye–RNA detection. In contrast, the sample containing a free mRNA reference standard in the placebo matrix showed a smaller relative change, indicating that free mRNA in a lipid nanoparticle matrix is less affected than mRNA formulated within LNPs. Although Triton X-100 disrupts the original LNP structure, it does not necessarily produce a true molecular solution. Differences in lipid content and composition between the samples may lead to the formation of distinct mixed lipid-surfactant micelles or larger assemblies, resulting in different effects on light scattering and fluorescence response. These results support the conclusion that LNP-associated optical and matrix effects can lead to underestimation of the fluorescence signal and should be carefully controlled by matrix matching and appropriate dilution during mRNA quantification.

This intact-versus-disrupted approach used for RiboGreen-based determination of mRNA concentration and EE% is convenient, but it relies on several assumptions. The detergent must efficiently disrupt the particles, the released mRNA must become available for dye binding, and the LNP matrix should not substantially alter the fluorescence response. Recent work has shown that RiboGreen-derived total mRNA concentration and EE% can depend on surfactant type, surfactant concentration, and LNP composition [14]. Thus, the assay readout may reflect not only RNA concentration, but also matrix effects introduced by the formulation and sample preparation [18]. This is consistent with broader concerns in LNP-mRNA analytics, where reproducibility and a clear definition of encapsulation-related parameters are important for meaningful comparison of formulations and assay outcomes.

This issue is important because LNPs do not actually form molecular solutions; rather, they form colloidal dispersions of lipid-rich nanoparticles in an aqueous medium. Their optical properties depend on particle size, concentration, aggregation state, polydispersity, and refractive index mismatch between the nanoparticle phase and the surrounding medium [19,20]. As a result, LNP dispersions may appear opalescent or turbid, especially at higher particle concentrations or in the presence of larger particles and aggregates. Colloidal particles scatter incident light, and scattering intensity depends on the properties of the dispersed phase. In nanoparticle suspensions, turbidity and light scattering are affected by particle size, morphology, concentration, polydispersity, and interparticle interactions [20,21]. In routine formulation work, these properties are often treated as appearance or, colloidal stability attributes. In fluorescence assays, these properties can become a direct analytical problem. Measurements can be affected at both excitation and emission stages. Scattering or optical attenuation at the excitation wavelength can reduce the amount of light reaching the dye–RNA complex, while attenuation at the emission wavelength can reduce or distort the fluorescence signal detected by the instrument. Turbid or optically dense samples may also contribute to inner filter effects, where excitation and/or emitted light is attenuated within the sample, leading to nonlinear fluorescence responses and biased concentration estimates [22,23]. These effects are especially relevant in plate-reader assays, where sample volume, optical path length, reading geometry, and particle distribution in the well can influence the measured signal.

Although nanoparticle-related assay interference is a known issue in optical and spectroscopic measurements, optical attenuation is unlikely to be the only source of bias. In RiboGreen measurements, several effects may occur simultaneously. Light scattering and inner filter effects can reduce the detected signal, while fluorescence quenching may alter the emission of the dye–RNA complex through interactions with nearby molecules, interfaces, surfactants, or nanoparticle surfaces [12,13,19,20]. The dye itself may also be affected by the lipid-containing matrix. LNP samples contain lipid interfaces, hydrophobic domains, PEG-lipid surface layers, ionizable lipids, and, after disruption, surfactants. These components may change dye availability or alter its fluorescence response before or during RNA binding, and, in case of UV–vis assays, may even directly interfere with measurement, as described by Nogueira et al. [10]. A study on lipid vesicle systems has shown that lipid composition can influence dye incorporation and fluorescence properties, supporting caution when interpreting quantitative fluorescence measurements in lipid-rich matrices [21].

RNA accessibility adds another important variable, particularly for EE% determination. In intact LNPs, the dye should detect only non-encapsulated, surface-associated, or otherwise accessible RNA. Encapsulated RNA should become detectable only after efficient disruption of the nanoparticle structure. If disruption is incomplete, RNA release is inefficient, or RNA remains associated with lipid components after surfactant treatment, total RNA may be underestimated and calculated EE% may be biased. Reports showing that surfactant type, surfactant concentration, and assay kinetics affect RiboGreen-derived mRNA loading and EE% indicate that RNA accessibility and matrix solubilization can directly influence the result [14,16]. In practice, these mechanisms are likely to overlap. A single mRNA-LNP sample can scatter light, attenuate excitation or emission, affect dye behavior, interact with assay reagents, and restrict dye access to RNA at the same time. The relative contribution of each effect may vary with formulation composition, particle size, concentration, aggregation state, dilution factor, surfactant conditions, and incubation time. The dependence of LNP performance on formulation composition is consistent with recent mRNA-LNP studies showing that changes in lipid composition can alter particle size distribution, surface charge, morphology, and transfection performance [24]. Thus, applying a one-size-fits-all approach to overcome analytical challenges in different LNP-based drug-delivery systems seems to be out of reach. Instead, we postulate a method-specific dilution factor taking into account both drug-delivery system composition and the purpose of quantitation, i.e., free versus encapsulated mRNA.

The main limitation of this study is the use of a single mRNA-LNP formulation in the experiments. Taking into account the physicochemical nature of the observed phenomenon, the recorded suppression may be generalized with a high degree of probability to other fluorescence methods for determination of mRNA. To overcome the lack of cross-validation of RiboGreen assay results against non-fluorescence-based assay, we analyzed the data based on signal intensity rather than on calculated mRNA concentration. Further studies are needed to better understand the individual contributions of optical attenuation, dye response, RNA accessibility, and nanoparticle disruption efficiency to the final fluorescence readout. Although turbidity and LNP-associated bias on RiboGreen-based mRNA quantification need further investigation, the timely reporting of initial research results is important to ensure the patient’s safety. 

Despite emergence of the novel UV–vis methods for mRNA quantification [9,11,25], their industrial application is severely limited by sensitivity and by potential interference from excipients or reagents used for sample preparation [10]. The novel methods for mRNA quantification need to have clear benefits to be incorporated for routine drug quality control, as changing the method’s documentation requires considerable effort. Thus, fluorescence-based methods, including the RiboGreen assay, are still widely used, which justifies further research on the optimizing experimental protocols. 

## 5. Conclusions

We have quantitatively assessed LNP matrix-associated optical and physicochemical effects on the fluorometric determination of mRNA concentration using the RiboGreen assay. Opalescent LNP dispersions affected the fluorescence readout in a concentration- and matrix-dependent manner, most likely through a combination of light scattering, optical attenuation within the excitation and emission wavelength ranges, and matrix-related effects on dye–RNA detection. The experiments revealed that the degree of sample dilution plays a critical role in the accurate mRNA quantification and may cause significant signal suppression (up to 19.42% under the conditions investigated). Also, free and encapsulated mRNA quantification is prone to different levels of bias. We postulate that a practical solution to ensure the reliability of analytical results is the application of a method-specific dilution factor during the sample preparation step before fluorescence detection. This study contributes to experimental advancement by using LNP-containing placebo, with the same lipid composition as in the studied drug-delivery system, as a model matrix causing turbidity. This study also highlights that reliable determination of mRNA concentration by the RiboGreen assay and accurate calculation of encapsulation efficiency requires careful control of LNP-associated bias to ensure the quality of the LNP-based drug-delivery systems and the patient’s safety. 

## Figures and Tables

**Table 1:** Summary statistics for analytical signal in samples containing a fixed amount of mRNA and different amounts of placebo.

	mRNA Standard (Without Placebo)	Placebo + TE (1:11, *v*/*v*)	Placebo + TE (1:7, *v*/*v*)	Placebo + TE (1:3, *v*/*v*)	Placebo + TE (1:1, *v*/*v*)	Placebo
n	30	6	6	6	6	6
Mean signal [RFU]	3402.2	3302.3	3309.9	3248.7	3083.0	2741.7
RSD [%]	2.36	1.19	1.12	0.36	0.97	0.55
Relative difference [%] *	-	−2.94	−2.71	−4.51	−9.38	−19.42
*p*-value	-	0.0003	0.0004	<0.0001	<0.0001	<0.0001
significance	-	S	S	S	S	S

* Relative difference between mean signals in sample with placebo and mRNA standard divided by mean signal for mRNA standard; S—significant (*p* < 0.05). TE = Tris-EDTA buffer.

**Table 2:** Summary statistics for analytical signal in samples with and without additional placebo (n = 6).

Parameter	Placebo	Placebo + mRNA Standard	LNP with mRNA	LNP with mRNA + mRNA Standard
Total mRNA concentration [µg/mL]	0	20	20	40
Mean signal [RFU] with additional placebo	39.3	2761.6	1441.5	3857.6
RSD [%]	1.29	1.41	1.45	1.39
Mean signal [RFU] without additional placebo	66.0	3573.6	3015.8	6275.2
RSD [%]	2.18	1.06	0.85	0.74
Relative difference [%] *	−40.46	−22.72	−52.20	−38.50
*p*-value	-	<0.0001	<0.0001	<0.0001
significance	-	S	S	S

* Relative difference between mean signals in sample with additional placebo and reference sample divided by mean signal for reference sample. S—significant (*p* < 0.05).

## Data Availability

The original contributions presented in this study are included in this article. Further inquiries can be directed to the corresponding author(s).

## Abbreviations

The following abbreviations are used in this manuscript:

CQAs	Critical quality attributes
EE	Encapsulation efficiency
IFE	Inner filter effect
LNPs	Lipid nanoparticles
mRNA	Messenger RNA
N/A	Not applicable
PBS	Phosphate-buffered saline
PDI	Polydispersity index
PEG	Polyethylene glycol
RFU	Reference fluorescence unit
RSD	Relative standard deviation
TE	Tris-EDTA
UV–Vis	Ultraviolet–visible

## References

[1] Liu L., Kim J.-H., Li Z., Sun M., Northen T., Tang J., Mcintosh E., Karve S., DeRosa F. (2025). PEGylated Lipid Screening, Composition Optimization, and Structure–Activity Relationship Determination for Lipid Nanoparticle-Mediated mRNA Delivery. Nanoscale.

[2] El-Mayta R., Padilla M.S., Billingsley M.M., Han X., Mitchell M.J. (2023). Testing the In Vitro and In Vivo Efficiency of mRNA-Lipid Nanoparticles Formulated by Microfluidic Mixing. JoVE.

[3] Ma Y., VanKeulen-Miller R., Fenton O.S. (2025). mRNA Lipid Nanoparticle Formulation, Characterization and Evaluation. Nat. Protoc..

[4] Liu Y., Huang Y., He G., Guo C., Dong J., Wu L. (2024). Development of mRNA Lipid Nanoparticles: Targeting and Therapeutic Aspects. Int. J. Mol. Sci..

[5] Nomani A., Saraswat A., Brown H., Kuo J., Duong H., Wu J., Zhang Y., Fu Y., Moon Y., Wahidi S. (2026). Identifying Key Factors Affecting mRNA-Lipid Nanoparticles Drug Product Formulation Stability. Nanomaterials.

[6] Schober G.B., Story S., Arya D.P. (2024). A Careful Look at Lipid Nanoparticle Characterization: Analysis of Benchmark Formulations for Encapsulation of RNA Cargo Size Gradient. Sci. Rep..

[7] Guerrini G., Mehn D., Scaccabarozzi D., Gioria S., Calzolai L. (2024). Analytical Ultracentrifugation to Assess the Quality of LNP-mRNA Therapeutics. Int. J. Mol. Sci..

[8] International Council for Harmonisation of Technical Requirements for Pharmaceuticals for Human Use (2023). ICH Q2(R2) Validation of Analytical Procedures.

[9] Misto S., Ferrillo T., Balog S., Quaglia F., Moore T.L. (2026). Comparison and Validation of a High-Throughput Lipid Nanoparticle Production and Characterization Workflow. Int. J. Pharm..

[10] Nogueira S.S., Samaridou E., Simon J., Frank S., Beck-Broichsitter M., Mehta A. (2024). Analytical Techniques for the Characterization of Nanoparticles for mRNA Delivery. Eur. J. Pharm. Biopharm..

[11] Le Ru E.C., Böttger R., Andrews D., Baumhof P., Rakonjac J.V., Laufersky G., Darby B.L. (2025). Rapid and Accurate Quantification of RNA in Lipid Nanoparticles by Scatter-Free UV/Visible Spectroscopy. Nano Lett..

[12] Jones L.J., Yue S.T., Cheung C.-Y., Singer V.L. (1998). RNA Quantitation by Fluorescence-Based Solution Assay: RiboGreen Reagent Characterization. Anal. Biochem..

[13] Carneiro S.P., Müller J.T., Merkel O.M., Astatke M. (2024). Fluorescent Techniques for RNA Detection in Nanoparticles. RNA Amplification and Analysis.

[14] Schultz D., Münter R.D., Cantín A.M., Kempen P.J., Jahnke N., Andresen T.L., Simonsen J.B., Urquhart A.J. (2024). Enhancing RNA Encapsulation Quantification in Lipid Nanoparticles: Sustainable Alternatives to Triton X-100 in the RiboGreen Assay. Eur. J. Pharm. Biopharm..

[15] Ong K.J., MacCormack T.J., Clark R.J., Ede J.D., Ortega V.A., Felix L.C., Dang M.K.M., Ma G., Fenniri H., Veinot J.G.C. (2014). Widespread Nanoparticle-Assay Interference: Implications for Nanotoxicity Testing. PLoS ONE.

[16] Bizmark N., Nayagam S., Kim B., Amelemah D.F., Zhang D., Datta S.S., Priestley R.D., Colace T., Wang J., Prud’homme R.K. (2024). Ribogreen Fluorescent Assay Kinetics to Measure Ribonucleic Acid Loading into Lipid Nanoparticle Carriers. Adv. Mater. Interfaces.

[17] Meredith S.A., Kusunoki Y., Evans S.D., Morigaki K., Connell S.D., Adams P.G. (2024). Evidence for a Transfer-to-Trap Mechanism of Fluorophore Concentration Quenching in Lipid Bilayers. Biophys. J..

[18] Guerrini G., Scaccabarozzi D., Mehn D., Sarracino A., Gioria S., Calzolai L. (2025). Challenges in Measuring In Vitro Activity of LNP-mRNA Therapeutics. Int. J. Mol. Sci..

[19] Parot J., Mehn D., Jankevics H., Markova N., Carboni M., Olaisen C., Hoel A.D., Sigfúsdóttir M.S., Meier F., Drexel R. (2024). Quality Assessment of LNP-RNA Therapeutics with Orthogonal Analytical Techniques. J. Control. Release.

[20] Anzini P., Redoglio D., Rocco M., Masciocchi N., Ferri F. (2022). Light Scattering and Turbidimetry Techniques for the Characterization of Nanoparticles and Nanostructured Networks. Nanomaterials.

[21] Balderas-Cabrera C., Castillo R. (2024). Mie Scattering Theory Applied to Light Scattering of Large Nonhomogeneous Colloidal Spheres. J. Chem. Phys..

[22] Friganović T., Weitner T. (2023). Reducing the Inner Filter Effect in Microplates by Increasing Absorbance? Linear Fluorescence in Highly Concentrated Fluorophore Solutions in the Presence of an Added Absorber. Anal. Chem..

[23] Xu J.X., Vithanage B.C.N., Athukorale S.A., Zhang D. (2018). Scattering and Absorption Differ Drastically in Their Inner Filter Effects on Fluorescence, Resonance Synchronous, and Polarized Resonance Synchronous Spectroscopic Measurements. Analyst.

[24] Fu J., Zhang Y., Lv Y., Li R., Gu H., Yang J. (2025). A Bioactive Lipid Nanoparticle Integrating Arachidonic Acid Enables High-Efficiency mRNA Delivery and Potent CAR-Macrophage Engineering. Int. J. Mol. Sci..

[25] López Espinar A., Le Ru E.C., Kumar P., Soares F., O’Driscoll C.M., Darby B.L., Kowalski P.S. (2025). Scatter-Free UV–Visible Spectroscopy for Accurate and Precise RNA Quantification in Complex RNA Nanoparticle Formulations. Anal. Chem..

